# Magnetic Sphincter Augmentation and Postoperative Dysphagia: Characterization, Clinical Risk Factors, and Management

**DOI:** 10.1007/s11605-019-04331-9

**Published:** 2019-08-06

**Authors:** Shahin Ayazi, Ping Zheng, Ali H. Zaidi, Kristy Chovanec, Nobel Chowdhury, Madison Salvitti, Yoshihiro Komatsu, Ashten N. Omstead, Toshitaka Hoppo, Blair A. Jobe

**Affiliations:** Esophageal and Lung Institute, Allegheny Health Network, 4815 Liberty Avenue, Suite 439, Pittsburgh, PA 15224 USA

**Keywords:** Gastroesophageal reflux disease (GERD), Dysphagia, Magnetic sphincter augmentation, Upper endoscopy, High-resolution manometry

## Abstract

**Introduction:**

Magnetic sphincter augmentation (MSA) results in less severe side effects compared with Nissen fundoplication, but dysphagia remains the most common side effect reported by patients after MSA. This study aimed to characterize and review the management of postoperative dysphagia and identify the preoperative factors that predict persistent dysphagia after MSA.

**Material and Methods:**

This is a retrospective review of prospectively collected data of patients who underwent MSA between 2013 and 2018. Preoperative objective evaluation included upper endoscopy, esophagram, high-resolution impedance manometry (HRIM), and esophageal pH testing. Postoperative persistent dysphagia was defined as a postoperative score of > 3 for the dysphagia-specific item within the GERD-HRQL at a minimum of 3 months following MSA. A timeline of dysphagia and dilation rates was constructed and correlated with the evolution of our patient management practices and modifications in surgical technique.

**Results:**

A total of 380 patients underwent MSA, at a mean (SD) follow up of 11.5 (8.7) months, 59 (15.5%) patients were experiencing persistent dysphagia. Thirty-one percent of patients required at least one dilation for dysphagia or chest pain and the overall response rate to this procedure was 67%, 7 (1.8%) patients required device removal specifically for dysphagia. Independent predictors of persistent dysphagia based on logistic regression model included (1) absence of a large hernia (OR 2.86 (95% CI 1.08–7.57, *p* = 0.035)); (2) the presence of preoperative dysphagia (OR 2.19 (95% CI 1.05–4.58, *p* = 0.037)); and (3) having less than 80% peristaltic contractions on HRIM (OR 2.50 (95% CI 1.09–5.73, *p* = 0.031)). Graded cutoffs of distal contractile integral (DCI), mean wave amplitude, DeMeester score, sex, and body mass index were evaluated within the model and did not predict postoperative dysphagia. Frequent eating after surgery, avoidance of early dilation, and increase in the size of the LINX device selected decreased the need for dilation.

**Conclusion:**

In a large cohort of patients who underwent MSA, we report 15.5% rate of persistent postoperative dysphagia. The overall response rate to dilation therapy is 67%, and the efficacy of dilation with each subsequent procedure reduces. Patients with normal hiatal anatomy, significant preoperative dysphagia, and less than 80% peristaltic contractions of the smooth muscle portion of the esophagus should be counseled that they have an increased risk for persistent postoperative dysphagia.

## Introduction

Gastroesophageal reflux disease (GERD) is the most prevalent foregut disease in the Western population.^1–3^ This disease is often a chronic condition and affects approximately 25% of the adult population in the USA and is the most common gastrointestinal indication for seeking medical attention worldwide. The two main treatment options for patients with GERD are long-term acid suppression therapy with proton-pump inhibitors (PPI) and laparoscopic fundoplication. Medical acid suppression therapy is an effective first-line therapy in most patients. However, nearly 40% of patients experience breakthrough symptoms.^4,5^ In addition, long-term use of PPI can lead to adverse events such as susceptibility to infectious diarrhea, osteoporosis, and drug interactions. Laparoscopic Nissen fundoplication is the surgical treatment option offered to patients whose condition has failed to respond to medical therapy or who desire to be free from dependence on medical therapy. However, this operation is underused due to the fears of long-term side effects such as gas bloat, inability to belch or vomit, and anatomic failure of the repair. The limitations of pharmacologic therapy and fundoplication leave many patients and clinicians in the difficult position to either tolerate a lifetime of drug dependence with incomplete symptom relief or to undergo a complex surgical procedure that is has been difficult to disseminate on a large scale and may have considerable side effects.

In 2012, the US Food and Drug Administration approved the use of magnetic sphincter augmentation (MSA) as a surgical intervention for the patients diagnosed with GERD. Since then, studies have shown that MSA is a safe and effective treatment resulting in freedom from PPI and pH normalization rates comparable with those reported for laparoscopic Nissen fundoplication.^5–8^ Magnetic sphincter augmentation has become an increasingly common procedure for patients with GERD and is currently offered to patients in more than 300 centers in the USA and approximately 30000 LINX devices (Ethicon, Johnson & Johnson, Shoreview, MN) have been implanted worldwide.

Although studies suggest that the side effect profile of MSA is superior to LNF as evidenced by less gas bloating and a preserved ability to belch and vomit,^9^ postoperative dysphagia remains the most common complaint of patients after MSA. Early postoperative dysphagia has been reported in 43–83% of the patients.^10–12^ Although dysphagia resolves in the majority of these patients after 8 weeks, this symptom persists in some patients and may require endoscopic dilation or device removal. Despite the higher prevalence of dysphagia after MSA, there is a paucity of data pertaining to the characterization and management of persistent dysphagia. More specifically, the factors that predict dysphagia after surgery have not been characterized. This study was designed to characterize dysphagia after MSA, review the management of this complaint, and identify the preoperative factors that predict persistent dysphagia after MSA.

## Methods

### Study Population

This is a retrospective review of prospectively collected data of patients who underwent MSA at Allegheny Health Network hospitals (Pittsburgh, PA) between 2013 and 2018. Approval was obtained from the Allegheny Health Network Institutional Review Board (IRB 2018-161) prior to the start of the study.

Inclusion criteria were symptomatic GERD patients 18 years or older with persistent GERD or laryngopharyngeal reflux symptoms despite maximal antisecretory therapy and objective evidence of reflux disease based on increased esophageal acid exposure on pH monitoring or a positive impedance-pH based on previously described criteria.^13–15^ Patients with a previous history of esophageal or gastric surgery, gross anatomic abnormalities such as esophageal stricture, significant esophageal dysmotility, or a known allergy to titanium were not included in this study. The presence of hiatal hernia was not a contraindication to MSA.

### Preoperative Assessment

All patients completed a detailed clinical evaluation with a focus on their foregut symptoms and acid suppression medication use, and completed the Gastroesophageal Reflux Disease-Health Related Quality of Life (GERD-HRQL) questionnaire while taking their usual dosing of antisecretory medication. The GERD-HRQL assesses GERD symptoms and patient satisfaction using a 0 to 5 rating scale. It is composed of 10 questions relating to the severity of heartburn, regurgitation dysphagia, odynophagia, and bloating.^16,17^ The total GERD-HRQL score is calculated by summing the responses to 10 questions with scores ranging from 0 to 50.^16^ Preoperatively, questionnaires were completed with subjects taking their usual dose of antisecretory therapy. Patients completed an objective foregut evaluation prior to consideration for surgery.

The routine preoperative objective assessment included the following tests:

1. Esophagogastroduodenoscopy (EGD) with biopsy: to assess the presence of esophagitis and Barrett’s esophagus, and the presence and size of a hiatal hernia.
2. High-resolution impedance manometry (HRIM): this test was performed using high-resolution manometry (4.2-mm diameter; Medtronic Inc., MN), equipped with 36 pressure transducers (1 cm apart), to assess the esophageal body peristalsis (organization and pressure) and upper and lower esophageal sphincter pressure, position, and length as previously described.^18^
3. Esophageal pH or impedance-pH monitoring: these tests were performed selectively using either Bravo pH monitoring (Medtronics, Shoreview, MN, USA) or multichannel intraluminal impedance (MII) pH monitoring (Sandhill Scientific Inc., Highlands Ranch CO).^15,19^ Prior to pH testing, proton-pump inhibitors were discontinued for 10 days. A DeMeester score > 14.7 was considered abnormal distal esophageal acid exposure. Impedance-pH testing was used in patients with predominate symptoms of laryngopharyngeal reflux with or without typical reflux symptoms using previously described criteria.^15^
4. Videoesophagram: this imaging study was done to evaluate gross pharyngeal and esophageal motility, and to further delineate the anatomy and assess for any potential mass or mucosal lesions, diverticulum, and to evaluate hiatal hernia and esophageal stricture or scarring.

### Postoperative and Outcome Assessment

Subjective postoperative outcomes were evaluated at routine visits at 2 weeks, 6 weeks, 6 months, and then yearly after surgery. Patients were assessed for resolution of their reflux symptoms, use of antisecretory medications, and procedure-related complications. Length of hospital stay, need for readmission within 90 days after surgery, and need for postoperative dilation and device removal were also recorded. Patients were asked to complete GERD-HRQL questionnaire at their 6-month and yearly visits. A 50% improvement in the total GERD-HRQL score compared with the baseline on antisecretory therapy was considered clinically significant in this study. Immediate dysphagia was defied as subjective postoperative dysphagia experienced within 8 weeks after surgery. Persistent dysphagia was defined as a postoperative dysphagia score > 3 on GERD-HRQL “difficulty swallowing” item at 3 months or later after MSA.

Baseline demographic, clinical, and objective foregut function test parameters were compared between patients with and without persistent postoperative dysphagia. Factors found to be statistically significant in this univariate analysis were entered into a logistic regression model to determine risk factors for persistent postoperative dysphagia. These results were then reported as odds ratios (OR) with 95% confidence intervals (CI). A timeline of dysphagia and dilation rates was constructed and correlated with the evolution of our patient management practices and modifications in surgical technique.

At 1-year following MSA, patients were approached for objective foregut evaluation using the same tests employed in the preoperative evaluation. A total of 172 patients completed Bravo pH monitoring and 81 patients underwent HRIM at their 1 year follow up.

### Surgical Procedure—Evolution and Current Practice

The LINX device (Ethicon, Johnson & Johnson, Shoreview, MN) consists of a series of titanium beads with magnetic cores hermetically sealed inside. The beads are interlinked with independent titanium wires to form a flexible and expandable ring with a “Roman arch” configuration. Each bead can move independently of the adjacent beads, creating a dynamic implant without limiting esophageal range of motion. The device is manufactured in different sizes, ranging from 13 to 17 beads, and is capable of nearly doubling its diameter when all beads are separated.

MSA is performed laparoscopically and consists of complete posterior mediastinal esophageal mobilization with restoration of intra-abdominal esophageal length (≥ 3 cm), interrupted posterior crural closure (without pledgets or mesh), and device placement at the level of the GEJ with the posterior vagus nerve trunk located on the outside of the magnetic ring. Early in our experience, a “minimal dissection” technique was used in patients with little to no HH; this approach did not include mediastinal esophageal dissection, the phrenoesophageal ligament was left intact, and there was no crural closure. A sizing procedure, which assesses esophageal circumference, is performed prior to selecting the size of the device. Earlier in our practice, we selected the size of the LINX device by increasing two beads from the point of release of the sizing device; we then changed our sizing protocol by increasing it to three beads from the point of release.

#### Dilation Technique

**Fig. 1:**
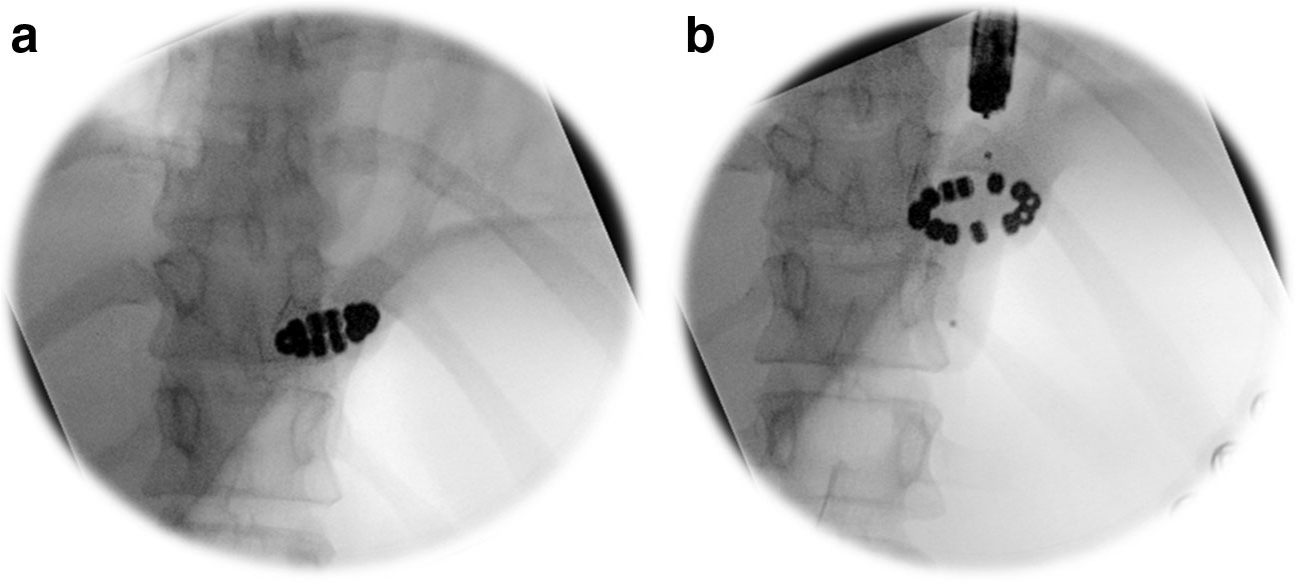
TTS balloon dilation under fluoroscopic guidance. **a** LINX device prior to dilation. **b** Separation of the titanium beads during dilation

Patients with dysphagia after MSA who did not experience improvement in their difficulty swallowing despite following dietary recommendations were considered for endoscopic evaluation and through-the-scope (TTS) balloon dilation under fluoroscopic guidance. The scope was advanced into the stomach. The balloon dilator (Boston Scientific CRE™, Natick, MA) was then inserted through the accessory channel of the endoscope and the scope was withdrawn to above the gastroesophageal junction to position the balloon within the magnetically augmented LES. Sequential dilation using 12–15 mm TTS balloon was performed under fluoroscopic guidance to assure separation of two-thirds of the titanium beads for approximately 30 to 60 s. If this was not achieved, a larger size balloon (15–18 mm) was used for dilation (Fig. 1).

### Statistical Analysis

Values are expressed as either mean with standard deviation (SD) or median with interquartile range (IQR) when appropriate. Statistical analysis was performed by means of nonparametric Mann–Whitney *U* test, Wilcoxon signed-rank test, and Pearson’s chi-square test when appropriate. Univariate analysis of the preoperative clinical and objective parameters that support persistent postoperative dysphagia risk was performed. The factors with *p* < 0.2 were included in a logistic regression model to produce the independent predictors for postoperative dysphagia. A *p* value < 0.05 was considered significant. Statistical analysis was performed using SAS software (SAS Institute Inc., Cary, NC).

## Results

A total of 380 patients underwent magnetic sphincter augmentation (MSA) during the study period. Demographic and baseline clinical characteristics of this cohort are shown in Table 1. At a mean (SD) follow up of 11.5 (8.7) months, 88.5% were satisfied with the outcome of surgery, 93.3% were free of PPI use, and 76.2% had normalization of their distal esophageal acid exposure. GERD-HRQL total score was improved from preoperative value of 33.7 (18.9) to 7.9 (9.8), *p* < 0.001.

**Table 1:** Baseline demographic and clinical characteristics

Characteristics	*N* (%)
Age (year)	
Mean (SD)	55.2 (13.6)
Gender	
Male	137 (36.1%)
Female	243 (64.0%)
BMI	
Mean (SD)	29.1 (4.5)
DeMeester score	
Mean (SD)	
*N* (%) with abnormal score (> 14.72)	293 (80.3%)
Esophagitis	
Yes	188 (49.5%)
No	192 (50.5%)
Size and type of Hernia	
None	45 (11.8)
Small (≤ 3 cm)	250 (65.8)
Large (≥ 3 cm)	66 (17.4)
PEH	19 (5.0)

Immediate postoperative dysphagia was reported by 240 (63.2%) of the patients. Persistent postoperative dysphagia was reported by 59 (15.5%) of patients, which was significantly lower than the preoperative baseline dysphagia rate of 35%, *p* < 0.001.

### Impact of Persistent Dysphagia on Outcome

Patients with persistent dysphagia were more likely to be unsatisfied with the outcome of surgery, to report less improvement in GERD symptoms, and to have a higher GERD-HRQL total score compared with those without persistent dysphagia. Although these patients had a higher rate of normalization of esophageal acid exposure, they were more likely to take PPI after MSA (Table 2).

**Table 2:** Comparison of outcome based on the status of postoperative persistent dysphagia

	Persistent dysphagia		*p* value
	No *n* (%)	Yes *n* (%)	
Total	321 (84.5%)	59 (15.5%)	
Satisfaction			
No	21 (6.7%)	22 (37.9%)	< 0.001
Yes	294 (93.3%)	36 (62.1%)	
GERD-HRQL total score			
Mean (SD)	6.8 (8.2)	22.8 (15.8)	< 0.001
GERD-HRQL heartburn score			
Mean (SD)	2.3 (4.5)	6.7 (8.3)	< 0.001
GERD-HRQL dysphagia score			
Mean (SD)	0.7 (0.8)	3.4 (0.7)	< 0.001
GERD clinical improvement			
No	45 (15.3%)	24 (46.2%)	< 0.001
Yes	249 (84.7%)	28 (53.8%)	
Normalization of acid			
Yes (DeMeester score < 14.7)	109 (73.7%)	22 (91.7%)	0.0546
No (DeMeester score ≥ 14.7)	39 (26.4%)	2 (8.3%)	
PPI use			
No	297 (94.6%)	49 (86.0%)	0.0169
Yes	17 (5.4%)	8 (14.0%)	

### Factors Contributing to Persistent Dysphagia

Demographic characteristics and the presence and degree of esophagitis or an abnormal DeMeester score were similar between those with and without persistent dysphagia. Lower esophageal sphincter resting and residual pressure, esophageal body mean wave amplitude, and distal contractile integral (DCI) were not different between the two groups.

**Fig. 2:**
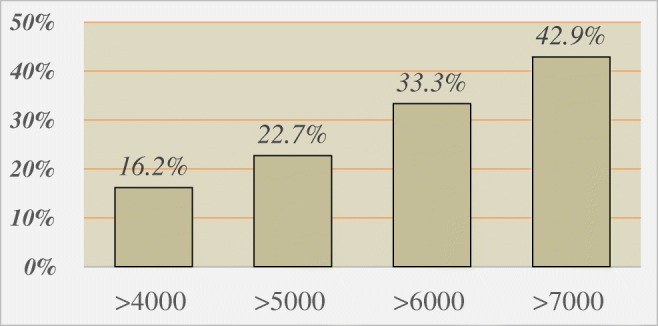
The prevalence (%) of persistent dysphagia among patients with hypercontractile esophagus, stratified using different DCI cutoffs showing a stepwise increase in the incidence of dysphagia in groups with higher DCI

Preoperative demographic, clinical, and physiologic parameters with potential contribution to the persistent postoperative dysphagia are shown in Table 3. Factors found to be relevant in this univariate analysis were used in a multivariable logistic model to determine predictors of persistent dysphagia. Absence of a large hernia, the presence of preoperative dysphagia, and having less than 80% peristaltic contractions on HRIM were found to be the three independent predictors of persistent dysphagia after MSA (Table 4). Graded cutoffs of distal contractile integral (DCI), mean wave amplitude, DeMeester score, sex, and body mass index were evaluated within the model and did not predict postoperative dysphagia. Although DCI did not prove to be significant within the logistic regression model, among patients with hypercontractile esophagus (DCI > 40000 mmHg cm sec), there was a stepwise increase in the rate of persistent dysphagia with an increase in the preoperative DCI (Fig. 2).

**Table 3:** Baseline potential predictors for persistent postoperative dysphagia adopting univariate logistic models

	Parameter (SE)	Odds ratio (95% CI)	*p* value
Age (< 50 years)	− 0.1372 (0.3044)	0.872 (0.480, 1.583)	0.6522
Gender (male)	0.3885 (0.3097)	1.475 (0.804, 2.706)	0.2098
BMI (< 30)	− 0.4573 (0.2853)	0.633 (0.362, 1.107)	0.1089
Presence of hiatal hernia	− 0.1277 (0.3348)	0.880 (0.457, 1.696)	0.7029
Absence of large or paraesophageal hernia	1.0588 (0.4498)	2.883 (1.194, 6.961)	0.0186
Esophagitis	− 0.3386 (0.2861)	0.713 (0.407, 1.249)	0.2366
Presence of grade C or D esophagitis	0.6132 (0.5459)	1.846 (0.633, 5.383)	0.2614
Abnormal preoperative DeMeester score (≥ 14.7)	0.0973 (0.3553)	1.102 (0.549, 2.212)	0.7841
Preoperative dysphagia	0.8451 (0.3426)	2.328 (1.190, 4.556)	0.0136
Elevated residual LES (> 15 mmHg)	− 0.3809 (0.4829)	0.683 (0.265, 1.760)	0.4302
Elevated LES resting pressure(> 43 mmHg)	− 0.6177 (0.4347)	0.539 (0.230, 1.264)	0.1553
Elevated intrabolus pressure (> 14.5 mmHg)	− 0.1530 (0.3202)	0.858 (0.458, 1.607)	0.6329
> 20% incomplete bolus clearance	0.4712 (0.4588)	1.602 (0.652, 3.937)	0.3044
Low DCI (< 500 mmHg s cm)	0.7527 (0.7531)	2.123 (0.485, 9.288)	0.3176
Peristalsis in < 80% of swallows	0.6969 (0.3998)	2.008 (0.917, 4.395)	0.0813
Low distal wave amplitude (< 40 mmHg)	− 0.7239 (0.6949)	0.485 (0.124, 1.893)	0.2976
Normal LES overall length (> 2.7 cm)	− 0.2087 (0.2890)	0.812 (0.461, 1.430)	0.4701
Normal intra-abdominal length (> 1.7 cm)	0.6280 (0.3475)	1.874 (0.948, 3.703)	0.0707
LINX size (≤ 13)	− 0.51 (0.31)	0.599 (0.330 1.090)	0.0934
LINX size (≤ 14)	− 0.56 (0.31)	0.573 (0.313, 1.051)	0.0720

**Table 4:** Independent predictors of persistent dysphagia using multivariable logistic model

	Parameter (SE)	Odds ratio (95% CI)	*p* value
Absence of large or paraesophageal hernia	1.05 (0.50)	2.86 (1.08, 7.57)	0.0346
Peristalsis in < 80% of swallows	0.92 (0.42)	2.50 (1.09, 5.73)	0.0306
Preoperative dysphagia	0.79 (0.38)	2.19 (1.05, 4.58)	0.0369

In a group of 81 patients who completed HRIM at their 1 year follow up, patients with postoperative dysphagia had significantly higher LES residual pressure (16.9 (6.9) vs. 12.0 (8.5), *p* = 0.031) and shorter intra-abdominal LES length (1.1 (1.3) vs. 2.5 (0.9), *p* = 0.049). There was also a trend toward higher DCI (3294.4 (1901.0) vs. 2328.5 (2124.1), *p* = 0.063) and higher mean distal wave amplitude (114.1 (36.2) vs. 93.0 (41.6), *p* = 0.081) in patients with persistent dysphagia compared with those without this complaint. Other manometric parameters on postoperative HRIM did not differ significantly between the two groups.

### Management of Postoperative Dysphagia

A total of 116 (30.5%) patients required at least one dilation for dysphagia or chest pain. The indication for dilation was dysphagia in 92 (24.2%), chest pain in 9 (2.4%), and both dysphagia and chest pain in 15 (3.9%).

A single dilation resolved the symptoms in (46/116) 39.6% of the patients. A total of 55 patients required second dilation with a response rate of (22/55) 40%, 23 required third dilation with resolution of symptoms in (8/23) 34.8%, nine patients had fourth dilation with a response rate of (2/9) 22.2%, and two patients underwent the fifth dilation and neither had response from the procedure. The overall response rate to dilation therapy (regardless of the number of dilations) was 78/116 (67%).

Twenty-four patients underwent dilation in the first 8 weeks following surgery, and only 4 (21%) of these patients had resolution of their dysphagia. Patients with dilation after 8 weeks had a significantly higher complete response rate (48.3% vs. 21%, *p* = 0.005). The comparison of dysphagia rates, need for dilation, and clinical and objective outcome stratified by the size of LINX used are shown in Table 5. There was a trend toward higher rate of immediate and persistent dysphagia in patients with a smaller device size used and more patients who had a smaller device placed required postoperative dilation. Fourteen patients in this cohort underwent explanation of their LINX. In 7 (1.8%) patients, the explanation was due to persistent dysphagia.

**Table 5:** Comparison of outcome, rate of dysphagia, and need for dilation by LINX size

	Device size					*p* value
	13 (*n* = 95)	14 (*n* = 134)	15 (*n* = 97)	16 (*n* = 38)	17 (*n* = 14)	
Immediate dysphagia (%)	68.4%	66.4%	55.6%	63.2%	50.0%	0.27
Persistent dysphagia (%)	21.1%	16.4%	11.3%	10.5%	7.1%	0.36
Dilation (%)	46.3%	38.1%	25.8%	34.2%	21.4%	0.03
Normalization of acid (%)	87.8%	78.0%	66.7%	75.0%	62.5%	0.087
GERD-HRQL total score, mean (SD)	9.4 (9.5)	10.6 (12.5)	8.0(11.2)	7.1(9.4)	10.1(15.5)	0.04

**Fig. 3:**
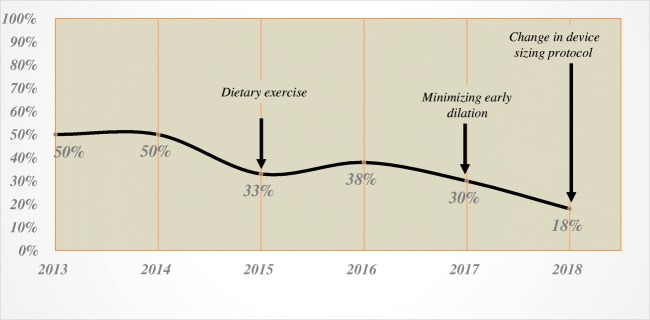
Prevalence (%) of the patients requiring dilation by year (2013–2018) and the impact of the changes in our patient management pathway and modification in the device sizing protocol on the rate of dysphagia

**Fig. 4:**
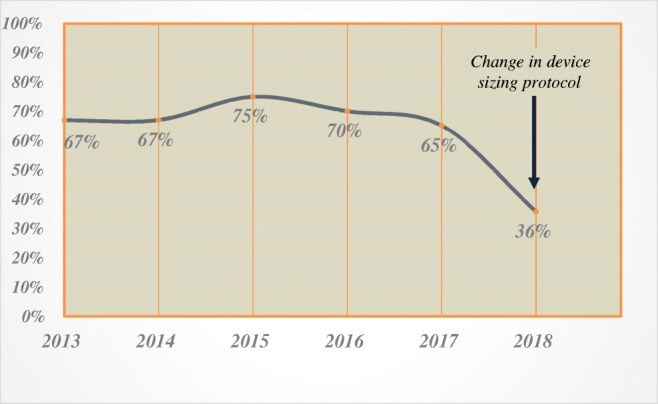
Prevalence (%) of the patients with immediate dysphagia after MSA by year (2013–2018) and the impact of modification in the device sizing protocol on the rate of immediate dysphagia

There was a temporal decrease in the need for dilation with the implementation of changes in our patient management pathway and modification of the device sizing protocol. More specifically, since we eliminated early dilation and instituted a strict diet protocol with the purpose of early and continuous device actuation, the dilation rate decreased from 50% in 2014 to 30% in 2017. Change in our sizing protocol to increase the size of the device by three beads from the point of release of the sizing device instead of two beads decreased the dilation rate even further to 18% in 2018 (Fig. 3). Similarly, we observed a noticeable decrease in the immediate postoperative dysphagia after change in our sizing protocol (Fig. 4).

## Discussion

Magnetic sphincter augmentation (MSA) was developed to address the need for an alternative treatment option in the management of patients with GERD through an outpatient laparoscopic procedure that does not alter gastric anatomy, augments the physiologic barrier to reflux, and can be reversed if necessary. MSA has gained popularity as a highly standardized technique for the treatment of reflux disease resulting in successful outcomes that are reproducible. This procedure is now offered in 300 centers in the USA and approximately 30,000 implants have performed worldwide.

Reflux disease and its complications are most often the consequence of an incompetent anatomical mechanical barrier. An ideal surgical option should restore this barrier with minimal to no side effects. The results of the feasibility, pivotal, and FDA post-approval studies have shown that magnetic sphincter augmentation is highly effective in reducing typical GERD symptoms, reducing daily PPI dependence, improving patients’ quality of life, and decreasing esophageal acid exposure.^12,20^ The results of the present study demonstrate a similarly high degree of symptom control, improvement in quality of life, and liberation from antisecretory medication use. MSA is comparable with LNF in the control of GERD symptoms while limiting procedure-related side effects,^12^ and therefore may provide a solution to the deficits experienced with the established medical and surgical GERD therapies.

Despite these advantages, persistent dysphagia in post-MSA patients ranges from 3 to 19% of cases, which is higher in contrast to post-fundoplication cases.^21^ In the present study, dysphagia was the most common procedure-related side effect seen in 15.5%. In the 2010 feasibility study of 44 patients who underwent MSA, early dysphagia was the most common complaint and occurred in 43% of the patients and in majority of patients resolved by 90 days, and in only one patient (2.3%), the device was explanted for persistent dysphagia.^22^ In the 2013 pivotal study of 100 patients who had MSA, dysphagia was again found to be the most frequent complaint, present in 68% of patients in the immediate postoperative period and reducing to 11% at 1 year and 4% at 3 years follow up.^23^ In the FDA post-approval study, the most common procedure-related symptom was dysphagia, present in 82.7% of the 67 patients but resolved in 79% of these patients at a median time of 8 weeks. A review of the FDA’s Manufacturer and User Facility Device Experience (MAUDE) data repository performed in 2016 revealed that 133 events deemed serious enough for report. In this report, dysphagia remained the most common complication with 60 cases submitted.^24^

Although studies indicate that dysphagia is the most common complaint after MSA, there is paucity of data in regard to characterization of the dysphagia, management of this complaint, and the factors that predict dysphagia after MSA. In this study, normal preoperative hiatal anatomy and dysphagia were found to be independent risk factors for persistent postoperative dysphagia. These findings likely reflect the complex nature of GERD from both anatomic and patient perception perspectives coupled with device sizing and the healing response at the GEJ after MSA; these factors are variable and unique to each patient. There is a higher degree of preoperative outflow resistance at the gastroesophageal junction in patients with hiatal hernia and surgical repair of hernia reduces this resistance, whereas those with normal preoperative hiatal anatomy may not have outlet obstruction from hiatal hernia and they may develop an amplified perception of postoperative dysphagia in the face of the relative obstruction caused with MSA. Preoperative dysphagia may result from an esophageal motility disorder, GERD-related inflammation, outlet obstruction from hiatal hernia, or a combination therein. The development of persistent postoperative dysphagia in this setting may represent a pre-existing predisposition to enhanced afferent neural input, combined with these preoperative clinical factors, which is then subsequently worsened by MSA. Despite having a lower mean postoperative DeMeester score, patients with persistent postoperative dysphagia had less improvement in the symptoms of GERD compared with those without dysphagia and were more often placed back onto antisecretory therapy. Postoperative esophageal distention from air swallowing driven by an increase in GEJ resistance after MSA is the probable culprit.

Currently, there are no established manometric criteria to guide patient selection for MSA. We showed that having less than 80% peristaltic contractions in the distal esophagus is a predictor of persistent postoperative dysphagia. This finding emphasizes the importance of consistent and organized peristalsis in the movement of a food bolus across the GEJ after the relative obstruction of MSA is imposed. This risk factor may serve as a harbinger for further degradation of effective peristalsis when the esophagus is subsequently challenged with MSA. Magnetic augmentation of the LES increases the outflow resistance of the GEJ. Therefore, the focus of the clinicians has been to not offer MSA in patients with weak esophageal motor function. However, less attention has been paid to those in whom an elevated DCI is indicative of vigorous esophageal contractility. Among patients with hypercontractile esophagus (DCI > 40000 mmHg cm sec), we observed a stepwise increase in the rate of persistent dysphagia with increasing DCI (Fig. 2) and these patients should be counseled that they may be at increased risk for persistent postoperative dysphagia.

The incidence of immediate postoperative dysphagia is 63% in our patients. This rate is consistent with the values reported in the literature and is higher than those reported for LNF. One partial explanation for this observation is that LNF patients remain on a liquid or soft diet for the first 7–10 days after surgery, whereas MSA patients are instructed to start solid food immediately after surgery. With improvement in our comprehensive preoperative counseling and management of patients’ expectations and detailed direction in postoperative diet, we observed a decline in the rate of immediate postoperative dysphagia. This decline, however, became more pronounced when we changed our protocol to increase device sizing (Fig. 4).

**Fig. 5:**
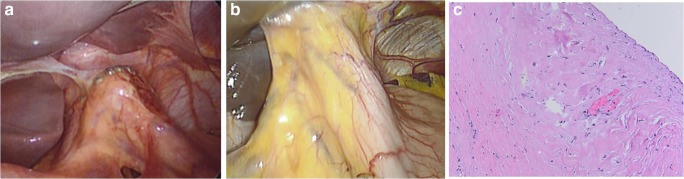
**a** Minimal fibrinous tissues deposition around the LINX at reoperation. **b** Dense fibrinous capsule and adhesions to liver after MSA. **c** Histopathologic examination of this capsule using hematoxylin and eosin staining shows dense deposition of collagen

The host response to the titanium magnetic ring is highly variable and may result in a spectrum of response pertaining to inflammation and healing; this variability most certainly impacts the dysphagia rates after MSA and will require further delineation and investigation. Our reoperative experience showed that within the first 6–8 weeks after MSA, we typically witness a loose network of tissues around the LINX (Fig. 5a), whereas later on, there is a robust capsule around the implant (Fig. 5b, c). While forming a robust fibrinous response around, the implant will have theoretical benefit of further augmentation of the reflux barrier and minimizing the chance of re-herniation, if there is exaggerated host inflammatory reaction, it may increase postoperative dysphagia. Anecdotally, we have observed such a response more often in those with underlying autoimmune or connective tissue disorders.

**Fig. 6:**
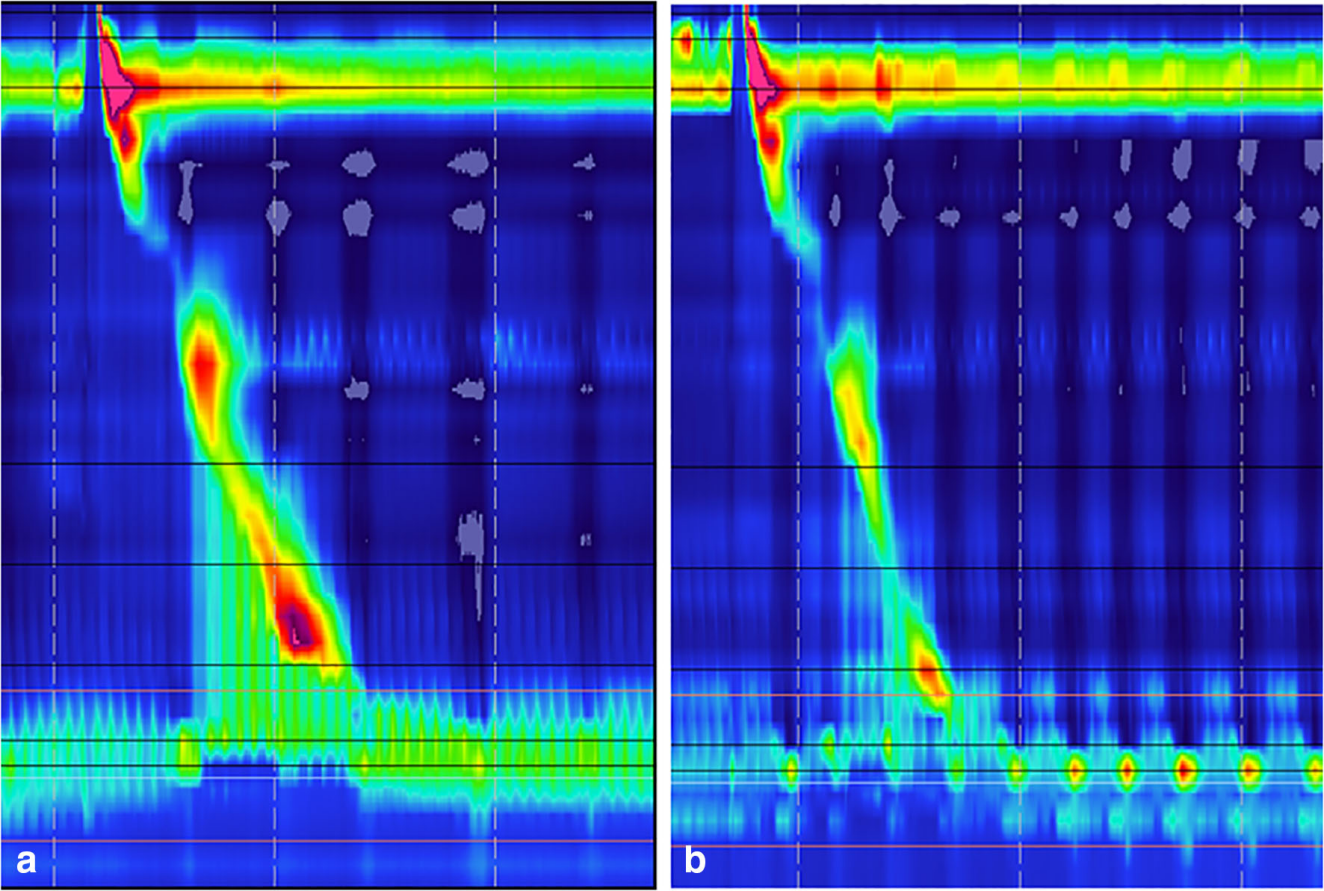
**a** HRM topographic tracing showing elevated intrabolus pressure (iBP) and outlet obstruction in a patient with significant dysphagia secondary to dense scarring or device undersizing. **b** HRM tracing of the same patient after explanation of the device with normalization of the iBP and relief of obstruction. Also note that GEJ augmented by MSA in **a** demonstrate a high-pressure zone with less pressure variability during the respiratory cycle, whereas after removing the implant in **b**, the variability in the high-pressure zone during the respiratory cycle becomes noticeable secondary to radial decompression

**Fig. 7:**
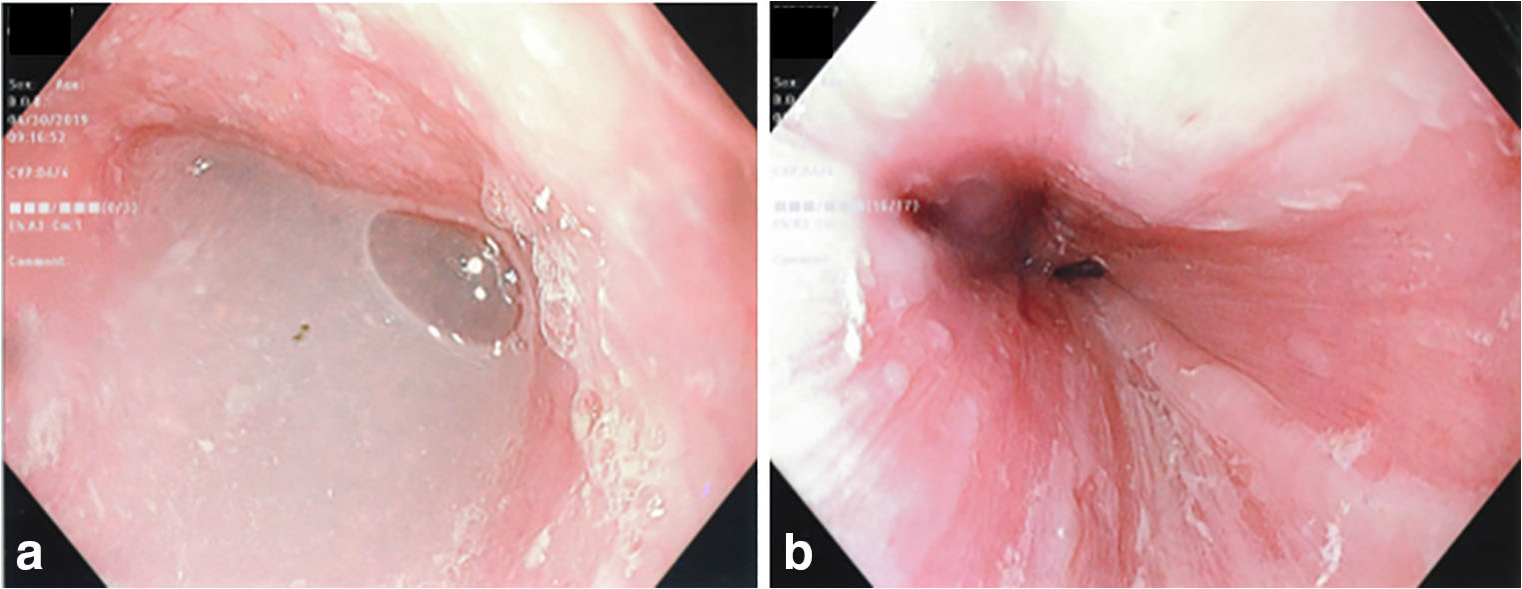
The endoscopic images of a patient with significant dysphagia showing **a** fluid retention above the GEJ and **b** esophageal lining changes suggestive for retention esophagitis

An increase in the outflow resistance caused by a tight crural closure or an undersized device that is evidenced by an increase in intrabolus pressure on HRM (Fig. 6a, b) or fluid retention and stasis esophagitis (Fig. 7) will only be remedied by addressing the anatomical problem. These findings emphasize the importance of detailed evaluation of the patients with persistent dysphagia after MSA with radiologic, endoscopic, or esophageal physiology testing.

As we gained experience with the management of postoperative dysphagia, we noticed the importance of three principles in decreasing the need for dilation after MSA:

- Eat a bite of solid food every hour for 8 weeks
- Avoidance of early dilation
- Increase in the size of the device selected

A solid diet is started on the day of surgery and patients are instructed to have frequent small meals and try to eat one tablespoon of yogurt or pudding or a cracker every hour while awake for 8 weeks after surgery. These dietary instructions facilitate device actuation and prevent scarring in the closed position. We have experienced a decrease in the rate of postoperative dysphagia and need for dilation after establishing these dietary recommendations.

Dilation within 8 weeks of surgery was not successful in alleviating dysphagia. In fact, many patients reported worsening of their dysphagia when the procedure was done in the early postoperative period. Early dilation can increase the acute inflammatory response around the device that can potentially worsen the dysphagia. If dysphagia persists beyond 8 weeks despite following dietary exercise, we then consider endoscopic dilation under fluoroscopic guidance followed by a 7-day course of steroid and 2 weeks of hourly eating.

In our practice, we prescribe 20 mg prednisone twice a day for 7 days with no tapering. We chose this regimen based on the review of literature and our clinical experience. This steroid course is not strong enough to suppress the hypothalamic–pituitary–adrenal (HPA) axis that would necessitate the need for steroid tapering, but it is enough to minimize the inflammatory response surrounding the implant. It is important to emphasize that there is currently no data to support the use of steroid after dilation in the relief of postoperative dysphagia. The current recommendations are based on expert opinion and high-volume center anecdotal experience.

**Fig. 8:**
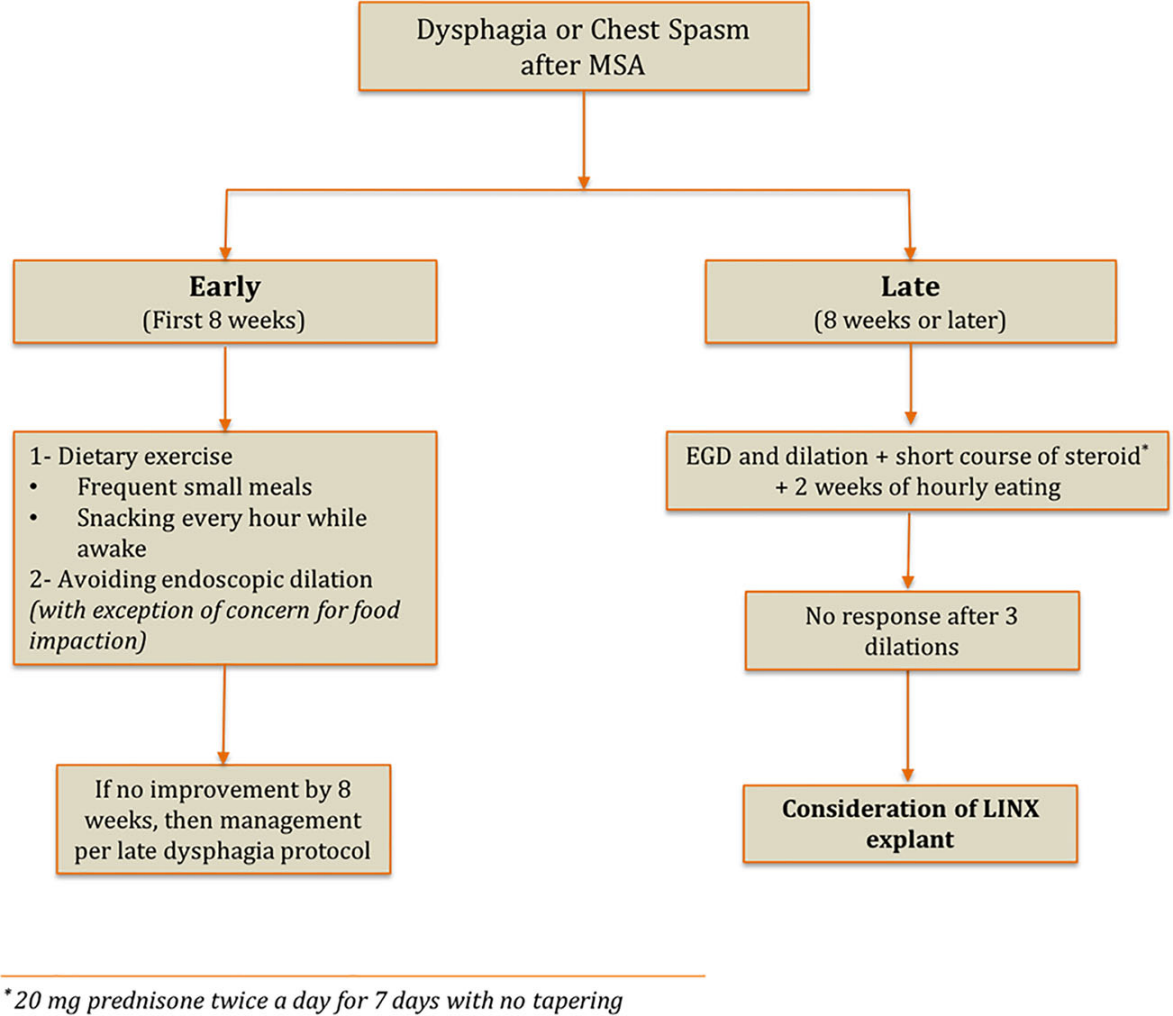
Proposed algorithm for management of postoperative dysphagia and chest spasm after MSA

There is reduction in the efficacy of dilation with each subsequent procedure, and after the third dilation, there is minimal benefit. We will consider device explant in patients with persistent dysphagia after three dilations. Our proposed algorithm in management of dysphagia after MSA is shown in Fig. 8.
